# Serum exosomal long noncoding RNA CRNDE level for hepatocellular carcinoma diagnosis

**DOI:** 10.1002/jcla.24144

**Published:** 2021-11-26

**Authors:** Xiao‐Li Huang, Guo‐Ming Zhang

**Affiliations:** ^1^ Department of Laboratory Medicine Sihong Hospital Sihong China; ^2^ Department of Laboratory Medicine Shuyang Hospital Shuyang China; ^3^ Department of Laboratory Medicine The affiliated Shuyang Hospital of Xuzhou Medical University Shuyang China

**Keywords:** CRNDE, diagnosis, hepatocellular carcinoma

Dear Editor,

Ting Wang et al.^1^ reported an article entitled “Serum exosomal long noncoding RNA CRNDE as a prognostic biomarker for hepatocellular carcinoma” in *Journal of Clinical Laboratory Analysis*. To evaluate the diagnostic efficiency of serum exosomal long noncoding RNA (IncRNA) CRNDE for hepatocellular carcinoma (HCC), the investigators collected 166 HCC patients and 100 healthy volunteers in this study. The serum exosomal lncRNA CRNDE was determined using qRT‐PCR, and the diagnostic performance of serum exosomal lncRNA CRNDE level by using receiver‐operating characteristic (ROC) curve (AUC) analysis in HCC. They found that serum exosomal lncRNA CRNDE level was significantly higher in HCC than normal controls and the AUC is 0.839. Therefore, they concluded that the serum exosomal lncRNA CRNDE was a diagnostic biomarker for HCC.

The study performed by Ting Wang et al. is very interesting for finding a non‐invasive biomarker for HCC. However, there are some limitations of this work. This work was not pre‐designed inclusion for enrolling study cohort consecutively, and the controls were 100 normal controls without HCC in this work. The design is a two‐gate design study^2^ in this work (the HCC and the controls come from two completely different cohorts. The number of HCC is crucial; if the number of HCC is too low, there will be lower estimated sensitivity, whereas if the number of normal controls is lower, there will be less and less estimate of sensitivity^2^). Therefore, the diagnostic capability of this work is strongly affected by the irrelevant controls and the number of normal controls.^3^, ^4^ The appropriate controls (including benign liver disease and normal controls) are crucial for evaluating serum exosomal lncRNA CRNDE level for HCC diagnosis. There are no risk factors, symptoms, and signs related to HCC for normal controls, and it is not necessary to determine serum exosomal lncRNA CRNDE to distinguish between normal controls and HCC. It should be using one‐gate design in this work (the HCC and the controls are from the same cohort. The number of HCC and the controls are fixed and constant. Therefore, the specificity and sensitivity of diagnostic tests will be constant, as long as the HCC and the controls come from the same cohort^2^) for better to use both exclusion criteria and pre‐specified inclusion for consecutively enrolling. In such a case, including patients with liver cancer, benign liver diseases, and non‐malignant diseases whose serum exosomal lncRNA CRNDE should be tested.

In conclusion, this work seems to provide serum exosomal lncRNA CRNDE as a non‐invasive diagnostic biomarker for HCC. However, due to the limitations of the study design. They need to use a one‐gate design for getting a well‐designed study. As we know, the controls should be included benign diseases and healthy people, and the number of controls is more than the number of cancer patients.

## Data Availability

The raw data supporting the conclusions of this manuscript will be made available by the authors, without undue reservation, to any qualified researcher.
